# Clinical Symptom Patterns as Predictors of SARS-CoV-2 Infection in Healthcare Workers in Puerto Rico

**DOI:** 10.3390/ijerph23010008

**Published:** 2025-12-19

**Authors:** Desiré Vázquez Ortiz, Josefina Romaguera, Jean L. Santos Agrait, Frances Vázquez, María E. Pérez, Carmen D. Zorrilla, Filipa Godoy-Vitorino

**Affiliations:** 1 Department of Mathematics, University of Puerto Rico, Rio Piedras Campus, San Juan, PR 00931, USA; desire.vazquez@upr.edu (D.V.O.); maria.perez34@upr.edu (M.E.P.); 2 Department of Obstetrics and Gynecology, School of Medicine, University of Puerto Rico, San Juan, PR 00918, USA; josefina.romaguera@upr.edu (J.R.); jean.santos6@upr.edu (J.L.S.A.); carmen.zorrilla@upr.edu (C.D.Z.); 3 Department of Microbiology and Immunology, University of Puerto Rico, Medical Sciences Campus, San Juan, PR 00918, USA; frances.vazquez5@upr.edu; 4 Comprehensive Cancer Center, University of Puerto Rico, Medical Sciences Campus, San Juan, PR 00921, USA

**Keywords:** COVID-19, Healthcare Workers (HCWs), cross-sectional study, General Logistic Regression (GLR), Receiver Operating Characteristic Curve (ROC)

## Abstract

**Highlights:**

**Public health relevance—How does this work relate to a public health issue?**

- SARS-CoV-2 infection among healthcare workers represents a critical occupational and public health concern due to their dual role as a high-risk group and potential sources of onward transmission within healthcare facilities and communities.
- This study addresses the need for evidence-based, symptom-driven screening strategies during periods of limited testing capacity by identifying clinical symptom patterns associated with SARS-CoV-2 positivity among healthcare workers in Puerto Rico.

**Public health significance—Why is this work of significance to public health?**

- The findings demonstrate that symptom burden and specific symptoms-particularly muscle pain, fever, and loss of taste or smell-are strong predictors of SARS-CoV-2 infection, highlighting practical indicators for early case identification in frontline workers.
- The model showed strong discriminative ability (AUC = 0.87).

**Public health implications—What are the key implications or messages for practitioners, policy makers and/or researchers in public health?**

- Symptom-based screening tools that prioritize key predictive symptoms and overall symptom count can guide testing prioritization and strengthen infection control measures in healthcare environments.
- Public health practitioners can use findings to refine occupational health protocols while researchers can build on this work to validate symptom-based models in larger and more diverse populations and during future outbreaks.

**Abstract:**

Coronavirus disease 2019 (COVID-19), caused by SARS-CoV-2, has posed major risks for healthcare workers (HCWs) worldwide. This study assessed the prevalence of infection and its relationship with demographic and clinical characteristics among HCWs at the University of Puerto Rico Adult Hospital. A total of 132 individuals were enrolled, of whom six tested positive (4.55%). The study population was predominantly female (78.8%) with a mean age of 41 years, and although men showed higher odds of infection (OR = 3.98), the difference was not significant. Symptom presence was strongly associated with infection: 7.4% of symptomatic participants tested positive compared to none of the asymptomatic (*p* < 0.001). Symptom count was also predictive, with those reporting three to four symptoms showing the highest positivity rate (14.8%) and those with five to ten symptoms at 6.7%. Specific symptoms including muscle pain (OR = 21.04, *p* = 0.002), taste loss (OR = 24.20, *p* = 0.002), smell loss (OR = 15.25, *p* = 0.024), and fever (OR = 20.50, *p* = 0.016) were significantly linked to infection, while others such as headache and congestion were not. These findings underscore the utility of symptom-based monitoring in occupational health, though the single-site design, modest sample size, reliance on self-report, and early pandemic diagnostic limitations may have led to underestimation of true cases.

## 1. Introduction

The COVID-19 pandemic, caused by the severe acute respiratory syndrome coronavirus 2 (SARS-CoV-2), has become a global public health crisis, with far-reaching impacts on healthcare systems worldwide. Healthcare workers (HCWs), as the frontline responders to the pandemic, are at a significantly higher risk of infection due to their proximity to infected patients, as well as their role in potential transmission to other patients and community members [1]. Early detection plays a key role in limiting transmission, as it is essential in maintaining the functionality of healthcare systems during such unprecedented times. However, despite their crucial role, HCWs remain at risk for both acquiring and transmitting SARS-CoV-2, with potential long-term consequences for their health and the broader population [2].

Several studies have highlighted the heightened vulnerability of HCWs, with reports showing increased rates of infection among this group compared to the general population [1,3]. However, the factors contributing to this increased risk remain poorly understood. Some research has suggested that the level of exposure, working conditions, and protective measures might influence infection rates among HCWs [4]. Notably, the difference in seroprevalence between occupations has varied in different studies [5]. In contrast, other studies have emphasized the importance of demographic factors such as age, comorbidities, and lifestyle in determining the susceptibility of HCWs to SARS-CoV-2 infection [6,7]. While the need for protective measures and effective infection control strategies is widely acknowledged, controversies persist regarding the best approaches to safeguard HCWs, especially in areas with limited healthcare resources.

It is important to distinguish between occupational SARS-CoV-2 exposure within healthcare facilities and non-occupational exposures occurring in the community. While occupational transmission can be mitigated through strict adherence to non-pharmaceutical interventions (masking, distancing, and hand hygiene), HCWs remained vulnerable to community-acquired infections, even in highly protected or vaccinated workforces [8]. This distinction is relevant when interpreting infection risk in HCWs. Prior research has explored the relationship between symptom burden and the likelihood of SARS-CoV-2 positivity, suggesting that the number of reported symptoms may serve as a potential indicator of infection. Although some reports have acknowledged the challenge posed by asymptomatic infection, numerous investigations have demonstrated that individuals with a higher symptom count and/or a higher symptom burden were significantly more likely to test positive for COVID-19, highlighting the diagnostic value of comprehensive symptom screening [9,10,11]. Notably, specific constellations of symptoms, such as fever, cough, and anosmia, have been particularly predictive of infection status in certain populations [12].

Our aim was to assess the prevalence of SARS-CoV-2 among HCWs at the University of Puerto Rico (UPR) Adult University Hospital, focusing on those directly involved in the care of COVID-19 patients. This study, based on a convenience sample from a larger microbiome study, also aimed to identify demographic, lifestyle, and health-related factors associated with an increased risk of infection, which could inform future prevention strategies. By establishing a population of high-risk individuals, this research contributes to the growing body of knowledge on SARS-CoV-2 transmission and the factors that influence its spread. Despite the extensive global research conducted on COVID-19, there remains a notable scarcity of data describing symptom-based predictors of SARS-CoV-2 infection among healthcare workers (HCWs) in Puerto Rico [13]. In the early stages of the pandemic, symptom screening was one of the primary strategies used in clinical settings to identify potential infections and reduce transmission risk. Gaining a clearer understanding of which symptom patterns are most strongly linked to SARS-CoV-2 positivity within the Puerto Rican HCW population is therefore critical for strengthening occupational health practices, especially in high-risk environments. This study helps fill that gap by examining the relationship between symptom count, specific symptoms, and infection status in a cohort of HCWs selected within the University of Puerto Rico healthcare system, offering insights that may guide future screening and local prevention efforts.

## 2. Methodology

### 2.1. Study Design and Recruitment

The umbrella study from which these HCW analyses were derived was designed to assess the prevalence of SARS-CoV-2 infection and oral microbiome among HCWs at the University of Puerto Rico (UPR). Participants were recruited using convenience sampling as part of a larger ongoing surveillance and microbiome project at the institution. The sampling for this substudy was conducted at the University District Hospital (UDH) from 20 July 2020 to 3 December 2020. Ethical approval for the study was granted by the Institutional Review Board (IRB) of the University of Puerto Rico School of Medicine (IRB protocol #B1760120), and approval for the biological samples was granted by the Institutional Biosafety Committee (IBC protocol #94320). All study procedures were conducted in accordance with ethical guidelines and principles of informed consent.

This study assessed SARS-CoV-2 prevalence among healthcare workers (HCWs) and university personnel not directly involved in patient care at the University District Hospital (UDH). A convenience sample of 132 individuals was recruited from various hospital departments, including physicians, nurses, residents, technicians, maintenance staff, administrative assistants, security personnel, social workers, medical laboratory scientists, and imaging staff.

Eligibility criteria included adults aged 21 to 80 years employed at UDH. Individuals younger than 21 or older than 80, as well as those with intellectual disabilities or mental health conditions that could impair informed consent, were excluded. Recruitment materials, including informational flyers, were distributed across UDH. Trained study personnel conducted the informed consent process in person, ensuring participants understood that involvement was voluntary and that they could withdraw at any time without penalty.

Following consent, participants completed a structured health and demographic questionnaire capturing socio-demographic characteristics, lifestyle behaviors, medical history (including chronic diseases, infectious conditions, and influenza vaccination), hygiene practices, and COVID-19 symptoms. Responses were initially recorded on paper and later entered into the REDCap system for management and analysis.

Sample collection included nasopharyngeal swabs for SARS-CoV-2 PCR testing, as well as saliva, oral swabs, anal/fecal swabs, and dried whole blood samples. These additional specimens were stored in a biorepository to support future microbiome, metabolomic, and cytokine analyses aimed at understanding biological changes associated with SARS-CoV-2 infection and immune response. All nasopharyngeal samples were collected at UDH and processed at the MSC Laboratory of Parasite, Immunology, and Pathology.

### 2.2. Sample Collection

Nasopharyngeal swabs were collected by trained HCWs at UDH for SARS-CoV-2 detection. In addition to nasopharyngeal samples, participants voluntarily provided additional biological samples for future microbiome analysis, including saliva, oral swabs, anal/fecal swabs, and whole blood. These samples were collected by participants themselves, except for nasopharyngeal swabs, which required healthcare personnel. All biological samples were transported to the MSC Laboratory of Parasite, Immunology, and Pathology for DNA/RNA processing and the virology Lab housed at the Microbiology Department for PCR using the CDC-006-00019 protocol.

### 2.3. Health and Demographic Questionnaire

Participants completed a detailed questionnaire that assessed socio-demographic characteristics, lifestyle factors, health status, and COVID-19-related symptoms. Key areas covered in the questionnaire included: Demographics: age, sex, employment status, household composition. Health and Medical History: chronic conditions, flu vaccination history, and any prior diagnosis of COVID-19. Lifestyle Factors: physical activity, smoking, alcohol consumption, and nutrition. COVID-19 Symptoms and Behavior: current and past symptoms, health behaviors during the pandemic (e.g., mask use, hand hygiene, social distancing). The questionnaire data were entered into REDCap (Research Electronic Data Capture) for secure data management and analysis. Smoking status was based on two questionnaire items asking whether participants had ever used cigarettes and whether they currently used them. Participants were classified as current smokers or non-smokers. Lifestyle variables included cigarette smoking and alcohol use. Alcohol consumption was based on yes/no questions regarding current use and categorized as a current alcohol user vs. a non-user. Physical activity and nutrition variables were listed in the questionnaire framework but were not collected in this dataset. Self-reported height and weight were used to calculate participants’ Body Mass Index (BMI). The instrument consisted of a structured, interviewer-administered questionnaire conducted in person by trained study staff during three scheduled encounters: at baseline (visit 1), three months (visit 2), and six months (visit 3). Most questionnaire items were closed-ended and adapted from standardized epidemiologic tools and early CDC COVID-19 symptom checklists. The research team developed the questionnaire specifically for this study, and it was not subjected to formal validation.

### 2.4. SARS-CoV-2 Detection

SARS-CoV-2 detection was performed using the CDC-006-00019 protocol. After RNA extraction from nasopharyngeal swabs, reverse transcription and quantitative PCR (qPCR) were performed with the TaqPath™ COVID-19 Combo Kit (Thermo Fisher Scientific, Inc., Waltham, MA, USA), following CDC guidelines (CDC-006-00019, Revision: 02). The assay utilizes primers and probes specific to SARS-CoV-2 targets, and the fluorescence intensity was monitored during each PCR cycle to determine the presence of the virus. A positive control was included in each batch, using RNA from the Human coronavirus 229E strain (ATCC^®^ VR-740D™). The PCR assay was performed in accordance with CDC guidelines, ensuring the specificity and sensitivity of the detection.

### 2.5. Follow-Up and Confirmatory Testing

Participants who tested positive for SARS-CoV-2 were contacted and asked to provide a confirmatory sample. Additionally, those with positive results were followed weekly until their COVID-19 tests returned negative. The results of these tests were provided via email, and participants were offered additional support for contact tracing and self-isolation.

### 2.6. Data Management

All data was securely stored and managed using the REDCap system. The data was anonymized to ensure confidentiality and was handled in accordance with ethical guidelines and principles of informed consent.

Data from the health and demographic questionnaire were analyzed to identify factors associated with SARS-CoV-2 positivity, including age, gender, health history, and lifestyle behaviors. Additionally, the relationship between BMI and COVID-19 symptoms was examined to explore potential risk factors for infection and morbidity

### 2.7. Metadata for Statistical Analysis

Data for this study was collected from workers, and the demographic and clinical characteristics of the participants were analyzed to assess associations with the likelihood of testing positive for SARS-CoV-2. The variables included in the analysis were sex, age, BMI, occupation, and symptom data (See Supplementary Table S1).

Participants were categorized by sex as either women or men. Age was divided into five groups: 21–30 years, 31–40 years, 41–50 years, 51–60 years, and 61–70 years. BMI (kg/m^2^) was classified according to NIH guidelines into normal (18.5–24.9), overweight (25–29.9), obese (30 or more), underweight (18.4 or less), and missing data categories. Occupation was categorized into four main groups: nurses, physicians, technicians, and others, including administrative assistants, security officers, social workers, medical laboratory scientists, and imaging center personnel.

Symptom data were also collected from the participants. They were categorized based on whether they reported symptoms or not. For those who reported symptoms, the number of symptoms was recorded, with participants being grouped into those reporting 1–2 symptoms, 3–4 symptoms, or 5–10 symptoms. In addition to the number of symptoms, specific symptoms related to COVID-19 infection were documented, including cough, shortness of breath, tiredness, diarrhea, fever, smell loss, taste loss, chills, muscle pain, headache, throat pain, vomiting, joint pain, chest pain, nasal congestion, sputum, finger/toe discoloration, stroke, conjunctivitis, tearing, and rash.

### 2.8. Statistical Analysis

Logistic regression was employed to analyze the relationship between various predictors and the likelihood of a positive SARS-CoV-2 test result. This method is appropriate for binary outcome data, enabling the estimation of the probability of a positive test result based on one or more predictor variables. The logistic regression model was fitted with the dependent variable being SARS-CoV-2 infection status (positive or negative) [14].

Univariate and multivariable logistic regression models were developed. The univariate model allowed us to examine the individual impact of each predictor (e.g., gender, age, occupation, and reported symptoms) on the likelihood of testing positive for SARS-CoV-2. This analysis provided insights into how each factor independently relates to the outcome. In the multivariable model, covariates such as age and the presence of reported symptoms were included to assess the combined effects of these variables while controlling for potential confounders. The adjusted odds ratios (ORs) and 95% confidence intervals (CIs) were calculated to clarify the relationships between predictors and SARS-CoV-2 positivity.

Additionally, the discriminative ability of the number of reported symptoms was evaluated as a predictor of test positivity. Fisher’s exact tests were initially used to assess the association between each individual symptom (e.g., cough, fever, muscle pain) and SARS-CoV-2 positivity. Subsequently, univariate logistic regression was conducted for each individual symptom, and odds ratios were computed. Two logistic regression models were then fitted to explore the relationship between the number of reported symptoms and the likelihood of testing positive: Model 1 assessed the general impact of symptom count, while Model 2 included both symptom count and age to examine whether age modifies the effect of symptom count on test positivity.

The final model’s predictive performance was assessed using Receiver Operating Characteristic (ROC) curve analysis. The area under the ROC curve (AUC) was calculated to evaluate the model’s ability to distinguish between positive and negative SARS-CoV-2 test results. A higher AUC value indicates better model performance. A *p*-value threshold of <0.05 was considered statistically significant.

### 2.9. Overview of Statistical Methods

For the analysis, a Generalized Linear Model (GLM) was employed to assess factors associated with the likelihood of testing positive for SARS-CoV-2 and the severity of symptoms. The Receiver Operating Characteristic Curve was used to evaluate the model’s discrimination ability. Data analysis was conducted using R version 4.0.3 [15] and the following R packages: glm from the base package for fitting generalized linear models, dplyr for data manipulation, ggplot2 for data visualization, pROC for receiver operating characteristic curve analysis, and tidyr for tidying data. These packages were used to process and analyze the data, including cleaning, transforming, and modeling the relationships between participant characteristics and COVID-19 outcomes.

To assess the factors associated with positive SARS-CoV-2 test results, logistic regression was employed. The analysis aimed to identify predictors of positive test results and explore the relationship between symptoms and test positivity. The primary outcome was the result of the SARS-CoV-2 PCR test, coded as positive (1) or negative (0).

### 2.10. Association Between Symptom Burden and Test Positivity

The impact of symptom burden was also explored on test positivity. Univariate logistic regression was conducted to assess the role of the total number of symptoms reported by each participant in predicting test results. In addition, a multivariable model was fitted, including both the number of symptoms and age, to examine whether age modifies the effect of symptom count on the likelihood of testing positive for SARS-CoV-2.

### 2.11. Model Validation and Predictive Performance

Fisher-exact tests were initially used to assess the association between each individual symptom and the likelihood of a positive SARS-CoV-2 test. Significant associations were further explored using logistic regression, providing ORs for each symptom. To assess the model’s discriminative ability, Receiver Operating Characteristic (ROC) curve analysis was performed. The area under the ROC curve (AUC) was 0.87, indicating strong discriminative power. This suggests that the logistic regression model can effectively distinguish between SARS-CoV-2 positive and negative test results based on the number of symptoms and other variables, despite the limited statistical power resulting from the small number of SARS-CoV-2 positive cases.

## 3. Results

### 3.1. Participant Characteristics

A total of 132 participants recruited from UDH were included in the analysis, of whom 6 tested positive for SARS-CoV-2, yielding a positivity rate of 4.55%. The demographic and clinical characteristics of the participants, along with their respective odds ratios (OR) and 95% confidence intervals (CI), are summarized below. The study population consisted of 28 males (21.1%) and 104 females (78.8%), with a mean age of 41.24 years. Among the participants, 61.4% reported experiencing COVID-19-related symptoms, as detailed in Table 1. A total of 6 individuals tested positive for SARS-CoV-2—3 men (10.71%) and 3 women (2.88%). Although the odds of testing positive were higher in men compared to women (OR = 3.98, 95% CI: 0.50–31.58), this difference was not statistically significant (*p* = 0.107). Regarding age, participants were divided into five groups: 38 individuals were aged 21–30 years, 2 of whom tested positive (5.26%); 31 participants were aged 31–40 years, with 3 testing positive (9.68%); 24 participants were aged 41–50 years, with 2 testing positive (4.17%); 30 individuals were aged 51–60 years, but none tested positive (0.00%); and 9 participants were aged 61–70 years, with no positive tests (0.00%). The differences in age were not statistically significant (*p* = 0.187). For BMI, 38 participants were classified as having a normal BMI, with only 1 positive case (2.63%). In comparison, 30 participants were overweight, with 3 testing positive (10.00%), yielding an odds ratio of 4.03 (95% CI: 0.31–221.25), although the result was not statistically significant (*p* = 0.462). Among obese participants (35 cases, 1 positive) and those with unavailable BMI data (26 cases, 1 positive), no significant differences were observed. In terms of occupation, nurses (50 participants) accounted for the majority of the sample, with 1 positive case (2.00%, reference group). Physicians (27 participants) had 3 positive tests (11.11%), with a lower odds ratio of 0.17 (95% CI: 0.00–2.21), while maintenance crew members, technicians, and others exhibited varied results.

### 3.2. Participant Symptoms

When considering the presence of symptoms, 81 participants reported experiencing symptoms, and 6 of these tested positive (7.41%). In contrast, among the 51 participants who did not report any symptoms, none tested positive (0.00%) (*p* < 0.001), indicating a significant association between symptom presence and test positivity.

In terms of the number of symptoms, participants who reported 3–4 symptoms (27 cases) had the highest positivity rate, with 14.81% testing positive. The odds ratio for this group was infinite (OR = Inf, 95% CI: 1.01–Inf), suggesting a strong association between the number of symptoms and the likelihood of testing positive. Similarly, those who reported 5–10 symptoms (15 cases) had a positivity rate of 6.67%, with an infinite odds ratio (OR = Inf, 95% CI: 0.50–Inf), further supporting the association between symptom count and test positivity.

Specific symptoms were also found to be significantly associated with SARS-CoV-2 positivity. For instance, muscle pain was strongly linked to positive test results, with an odds ratio of 21.04 (95% CI: 3.20–413.19, *p* = 0.002). Similarly, taste loss (OR = 24.20, 95% CI: 3.72–165.47, *p* = 0.002), smell loss (OR = 15.25, 95% CI: 1.76–108.84, *p* = 0.024), and fever (OR = 20.50, 95% CI: 2.25–165.21, *p* = 0.016) were also significantly associated with positive test results. Other symptoms, such as tiredness, throat pain, cough, diarrhea, nasal congestion, and chills, showed weaker associations.

It is important to note that “Other” occupations included administrative assistants, security officers, social workers, medical laboratory scientists, and imaging center personnel. Additionally, “Inf” refers to an infinite odds ratio, indicating that no events occurred in the comparison group, while an “Inf” in the 95% confidence interval indicates that the upper bound could not be calculated due to perfect separation in the data.

### 3.3. Association Between Symptoms and SARS-CoV-2 Status

An analysis of the number of symptoms reported by participants was conducted to evaluate its association with SARS-CoV-2 positivity. The results showed a significant difference in the number of symptoms between participants who tested positive and those who tested negative for SARS-CoV-2, with a *p*-value of less than 0.001 (Table 1). This highly significant result indicates a strong association between the number of symptoms reported and the likelihood of testing positive for SARS-CoV-2, suggesting that individuals with a higher number of symptoms are more likely to be infected with the virus. This finding highlights the importance of symptom monitoring in identifying individuals at higher risk of SARS-CoV-2 infection.

In addition to the total number of symptoms, specific symptoms were also found to be significantly associated with SARS-CoV-2 positivity. The symptoms with the most significant associations were: Muscle pain, with a *p*-value of 0.002; Taste loss, with a *p*-value of 0.002; Smell loss, with a *p*-value of 0.024; and Fever, with a *p*-value of 0.016 (Table 1).

These results suggest that individuals who reported these symptoms were significantly more likely to test positive for SARS-CoV-2, highlighting the importance of these symptoms as potential indicators of infection. Notably, muscle pain, taste loss, and fever exhibited particularly strong associations, with *p*-values below the commonly accepted threshold for statistical significance (*p* < 0.05).

### 3.4. Symptom Count and SARS-CoV-2 PCR Positivity

#### 3.4.1. Model 1: Symptom Count Alone

**Figure 1:**
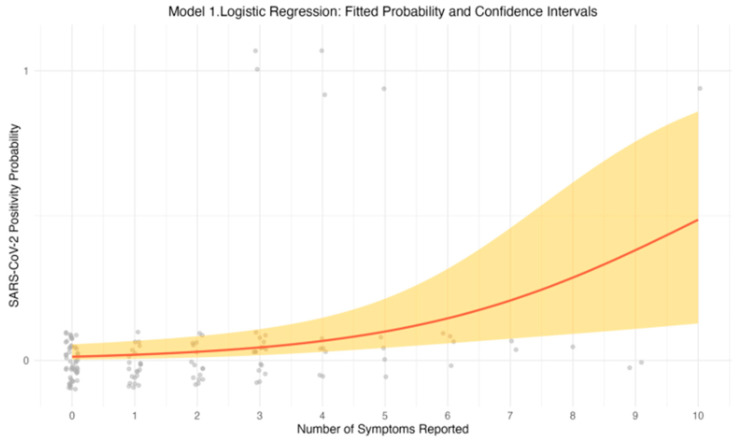
Predicted probability of testing positive for SARS-CoV-2 (red line) as a function of the number of symptoms reported. The shaded region represents the 95% confidence interval for the predicted probabilities, and the dark gray dots represent the observed data points.

The univariable logistic regression model revealed a positive association between the number of reported symptoms and the likelihood of testing positive for SARS-CoV-2. As the number of symptoms increased, the odds of a positive PCR result also increased. Specifically, individuals reporting a greater number of symptoms had higher odds of testing positive, suggesting that symptom count is a strong predictor of SARS-CoV-2 infection. This relationship is further illustrated in Figure 1, which depicts the predicted probability of testing positive for SARS-CoV-2 as a function of symptom count. The upward trend in the graph indicates that with each additional symptom, the probability of a positive test result increases. The fitted curve, along with the 95% confidence intervals (CIs), emphasizes the strength of this association.

#### 3.4.2. Model 2: Symptom Count and Age

**Figure 2:**
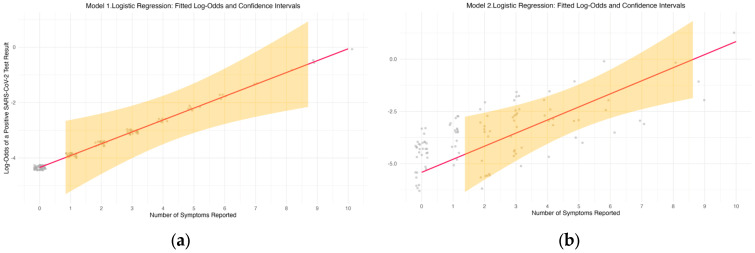
Log-odds (red line) of testing positive for SARS-CoV-2 as a function of the number of symptoms reported, as estimated from a logistic regression model. The plot illustrates the relationship between the number of symptoms and the log-odds of a positive test result. (**a**) Unadjusted general logistic regression (Model 1), (**b**) Adjusted general logistic regression (Model 2). Gray dots represent individual participant’s reported symptoms counts, and the yellow shading indicates the 95% confidence interval (CI).

When age was added as a covariate, the relationship between symptom count and PCR positivity remained significant. This indicates that the number of symptoms is a robust predictor of SARS-CoV-2 positivity, independent of age. The inclusion of age in the model allowed for a more nuanced understanding of how age might influence the effect of symptom count on test outcomes. The log-odds plot in Figure 2b illustrates the predicted log-odds of testing positive as a function of symptom count, with age considered as a covariate. Log-odds refer to the natural logarithm of the odds of an event occurring. In this case, the “odds” represent the likelihood of testing positive for SARS-CoV-2. A positive log-odds value indicates an increased likelihood of testing positive, while a negative value indicates a decreased likelihood. The plot demonstrates that while the effect of symptoms on test positivity increases with age, the influence of symptom count on the likelihood of a positive result diminishes as the number of symptoms reported becomes larger. The plot demonstrates that while the effect of symptoms on test positivity increases with age, the influence of symptom count on the likelihood of a positive result diminishes as the number of symptoms reported becomes larger.

To evaluate whether age acted as a cofounder in the association between the number of symptoms and SARS-CoV-2 positivity, we compared an unadjusted logistic regression (Model 1) with an additionally adjusted model for age (Model 2). The inclusion of age did not materially strengthen the model (see Table 2). The symptom coefficient changed only minimally after adjustment, and the fitted log-odds curves and their confidence intervals remained largely overlapping between the adjusted and unadjusted models (see Figure 2). This lack of improvement may reflect limited statistical power, given the small number of SARS-CoV-2 positive cases.

**Figure 3:**
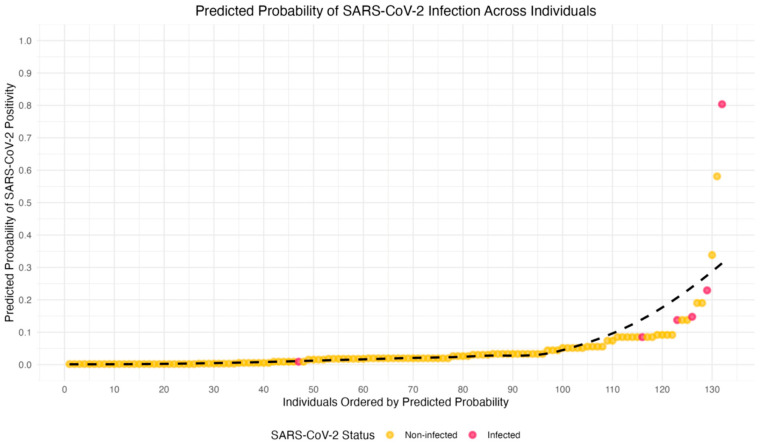
Predicted probabilities of testing positive for SARS-CoV-2 based on logistic regression analysis (dash line). Each point represents an individual’s predicted probability, with yellow dots indicating participants who tested negative and red dots indicating those who tested positive.

Figure 3 provides a graphical representation of the predicted probabilities of SARS-CoV-2 infection, based on the logistic regression models. The individuals are ranked by their predicted likelihood of testing positive for SARS-CoV-2, with probabilities ranging from 0 (low probability) to 1 (high probability).

In this visualization, the *x*-axis represents the rank order of individuals by their predicted probability of infection, and the *y*-axis shows the actual predicted probability of testing positive. Each point corresponds to an individual HCW, with points colored red for those who tested positive and yellow for those who tested negative. A LOESS-smoothed curve (dashed line) is added to highlight the overall trend in the relationship between predicted probabilities and actual test outcomes. This plot demonstrates that individuals with higher predicted probabilities are more likely to test positive for SARS-CoV-2, and provides a clear visual representation of the variability in predicted probabilities across individuals, even among those with the same test outcome. The graph underscores the complexity and uncertainty of predicting SARS-CoV-2 infection.

**Figure 4:**
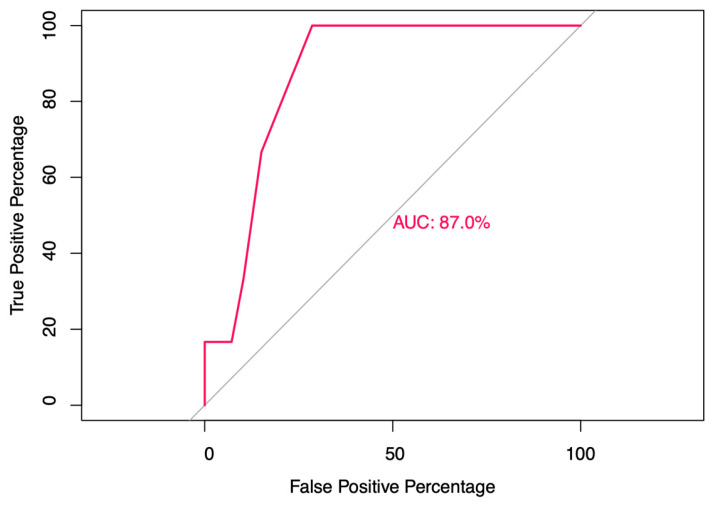
Receiver operating characteristic (ROC) curve analysis is displayed in percentage scale, demonstrating good discriminative performance with an AUC of 87%. The diagonal gray line represents the reference line corresponding to an AUC of 50%, extending from the origin (0, 0) to (100, 100).

A receiver operating characteristic (ROC) analysis was performed to evaluate the ability of symptom count to discriminate SARS-CoV-2 positivity. The model achieved an area under the curve (AUC) of 0.87, indicating good discriminative performance (see Figure 4). However, the estimate should be interpreted with caution due to the small number of positive cases (n = 6), which may affect the stability of the ROC curve.

The analysis reveals that symptom count is a significant predictor of SARS-CoV-2 positivity, with each additional symptom increasing the likelihood of a positive test result. The inclusion of age as a covariate strengthens the model and provides deeper insight into how symptoms count and how age interacts to influence the likelihood of infection. Furthermore, the graphical representations help visualize the predictive power of the model, illustrating both the overall trend and the variability in predicted probabilities. These findings reinforce the utility of symptom-based screening tools for early identification of COVID-19 in healthcare environments.

## 4. Discussion

### 4.1. Interpretation of Key Findings

Our analysis demonstrated that an increase in the number of symptoms reported by participants was strongly associated with an increased likelihood of a positive SARS-CoV-2 PCR test result. Specifically, individuals with a higher number of reported symptoms had significantly higher odds of testing positive for the virus, as evidenced in both univariate and multivariable logistic regression models. This finding aligns with previous studies that have highlighted symptom count as a reliable predictor of SARS-CoV-2 positivity [7,9].

Specific symptoms, such as muscle pain, taste loss, smell loss, and fever, were observed to be significantly associated with SARS-CoV-2 positivity, further emphasizing the importance of these symptoms in identifying individuals at higher risk for infection. Our results suggest that symptom monitoring, particularly for these key symptoms, could be valuable in early identification and isolation of individuals who may be infected with SARS-CoV-2.

When controlling for age, the relationship between symptom counts and PCR positivity remained minimally significant. This suggests that while age may influence susceptibility to SARS-CoV-2, the number of symptoms remains a crucial factor in determining the likelihood of infection. Notably, the inclusion of age in the model provided insights into how age may modify the effect of symptom count, with older individuals appearing to have an increased risk when they report multiple symptoms. However, the impact of symptom count remained robust across different age groups, reinforcing its predictive value independent of age.

### 4.2. Comparison with Previous Research

Our findings are consistent with several studies that have emphasized the role of symptom severity and symptom count in predicting SARS-CoV-2 infection. For instance, a study by Menni et al. [16] demonstrated that individuals with a higher symptom burden were more likely to test positive for SARS-CoV-2, which corroborates our own results. Similarly, Rahman et al. [17] found that symptom count, particularly in the early stages of infection, could serve as an effective screening tool for identifying high-risk individuals.

However, our study also extends previous research by highlighting age as a modifying factor in the symptom-infection relationship. While age has been shown to influence COVID-19 severity and outcomes [17], its interaction with symptom count in determining the likelihood of testing positive has not been widely explored. Our results suggest that older individuals with a higher number of symptoms are particularly at risk, which may inform more targeted screening strategies in healthcare settings.

Furthermore, our findings also shed light on the critical role of specific symptoms in predicting SARS-CoV-2 infection. Similar to previous studies, muscle pain and taste loss emerged as particularly strong predictors [18,19], with individuals reporting these symptoms showing substantially higher odds of testing positive for SARS-CoV-2. Specifically, those with muscle pain had 21 times greater odds (95% CI: 3.20, 413.19) of a positive PCR result, while individuals with taste loss had 24 times greater odds (95% CI: 3.72, 165.47) (Table 1). These findings are significant, as they suggest that these symptoms should be prioritized during initial screenings, particularly in high-risk settings, where they offer a higher predictive value for identifying individuals at risk of infection.

Several common COVID-19 symptoms such as fatigue, insomnia, and concentration difficulties are highly nonspecific and may overlap with mental-health–related manifestations, including anxiety or depression [20]. As noted in post-acute COVID-19 research, symptom screening tools may help reduce false positives by distinguishing infection-related symptoms from nonspecific complaints [21]. In contrast, symptoms such as headache and nasal congestion did not exhibit significant associations with PCR positivity, which suggests that these symptoms alone may be less reliable indicators of COVID-19 infection. This highlights the importance of considering a comprehensive set of symptoms, rather than relying on individual manifestations, in the early assessment of potential COVID-19 cases. Our results support the growing consensus that symptom count, rather than isolated symptoms, is a more effective predictor of COVID-19 infection [7,9]. Although the study reflects the pre-vaccine phase, its findings remain relevant for understanding symptom-based triage in future respiratory outbreaks and in post-vaccine settings where breakthrough infections may present atypically.

Compared to US and Brazilian HCW cohorts, the symptom profile observed in Puerto Rico showed similar associations with fever, myalgia, and sensory changes (anosmia/ageusia), but lacked several predictive features described elsewhere. In the United States, headache and malaise were significant predictors of infection and dyspnea was reported more frequently among positives, whereas nasal symptoms were inversely associated with PCR positivity [22]. In contrast, Brazilian HCWs more commonly exhibited nasal congestion, rhinorrhea, odynophagia, and gastrointestinal symptoms, with dyspnea notably absent among positive cases [23]. These regional differences highlight the heterogeneity of symptom expression across populations.

### 4.3. Implications for Healthcare Workers and Public Health

Although the study was conducted during the acute pandemic period (2020), its findings remain relevant for understanding early infection patterns among HCWs. Our findings suggest that symptom monitoring could be an effective strategy for identifying HCWs who may be at higher risk of testing positive, even in the absence of confirmed exposure. Given the nature of healthcare settings, where symptomatic individuals are likely to encounter vulnerable patients, early detection of potential infections among HCWs is critical for preventing outbreaks and protecting both HCWs and patients.

The relationship between symptom counts and infection also has broader public health implications. It may be used to refine screening protocols in high-risk settings, particularly in regions like Puerto Rico, where the healthcare system faces significant strain due to the ongoing pandemic. As symptom-based screening becomes more widespread, understanding the factors that influence symptom severity—such as age and underlying health conditions—can aid in prioritizing testing and protective measures for those most at risk. Our study highlights the value of integrating symptom-based assessments with clinical history to improve the early detection and management of SARS-CoV-2 cases.

### 4.4. Study Limitations

Although our study provides valuable insights into the relationship between symptom count and SARS-CoV-2 infection, there are several limitations to consider. First, the study was conducted in a single healthcare setting, which may limit the generalizability of the findings to other regions or populations. HCW might have been using better protective barriers than the general population, which could have affected both the infection rates and the potential viral loads leading to symptoms. The sample size, while adequate for exploratory analyses, was relatively small, and a larger study population would help validate these findings and assess the robustness of the associations identified. The questionnaire used for the sample collection in this study was not formally validated.

Furthermore, the cross-sectional nature of the study means causality cannot be inferred from the observed associations. Longitudinal studies would be necessary to explore the temporal dynamics of symptom development and infection. Another limitation is the reliance on self-reported data for some of the symptoms, which could introduce reporting bias. Because symptoms were self-reported, they are also subject to recall bias and misclassification. Nonspecific symptoms, such as fatigue, insomnia, or concentration difficulties, may reflect unrelated conditions, potentially resulting in over-reporting among some individuals. Although a standardized questionnaire was used in an attempt to mitigate this, self-reported symptoms may not fully capture the severity or accuracy of the symptoms experienced.

Additionally, the study did not assess viral load or other biomarkers that may influence test results, which could provide a more nuanced understanding of the relationship between symptoms and PCR positivity. Future research should consider incorporating these factors to further elucidate the mechanisms underlying symptom development and infection risk.

### 4.5. Epidemiological Profile of COVID-19 in Puerto Rico

Since the first cases of COVID-19 in March 2020, Puerto Rico has experienced multiple epidemic waves shaped by variant emergence, population immunity, and public health interventions. The COVID-19 pandemic in Puerto Rico followed epidemiological patterns that corresponded to initial widespread decreases in cases following lockdowns, and subsequent increases as restrictions eased. These patterns corresponded with the timing of the sampling for this study at a time when vaccines were not yet available.

The Epidemic status report by the PR Department of Health shows moderate peaks (10–49.9/100,000 population) from March to June 2020, followed by significant increases (50–99.9/100,000 population) in June and a rise to >100/100,000 population from August to December 2020 [24]. The epidemiological curves for reported cases of COVID using molecular testing showed a peak in March 2020 with the Alpha variant (British strain), followed by rises in September and December 2020 [25]. The specific data presented here was developed while Puerto Rico was still actively managing the pandemic and while diagnostic methods were being refined. It is possible that in symptomatic individuals with lower viral loads, SARS-CoV-2 infections were missed, leading to underestimation of infection rates. HCW had access to protective measures while at work, which might have affected both the positivity rates and the viral loads of SARS-C0V-2 in our sample.

## 5. Conclusions

Our findings highlight the predictive value of specific symptoms, particularly muscle pain and taste loss, in identifying SARS-CoV-2 infection among healthcare workers. These symptoms showed strong associations with PCR positivity and should be prioritized in screening protocols, especially in high-risk clinical settings. Symptom count also emerged as an important independent predictor, with the likelihood of testing positive increasing as the number of reported symptoms rose, regardless of age. This underscores the utility of considering both the presence and burden of symptoms in assessing infection risk. Taken together, these findings underscore the importance of symptom-based screening in identifying individuals at risk for SARS-CoV-2 infection, especially in healthcare settings where early detection is crucial. By prioritizing symptoms like pain and taste loss, healthcare providers can more effectively allocate testing resources, protect vulnerable populations, and reduce the spread of COVID-19. Moving forward, further research is needed to validate these findings across larger and more diverse populations, as well as to explore how symptom-based screening can be integrated into broader public health strategies to combat the ongoing pandemic.

These findings should be interpreted considering several limitations, including the small sample size, single-site setting, and reliance on self-reported symptoms, which may introduce recall bias or misclassification. Additionally, because the analysis was cross-sectional and based on early-pandemic symptom checklists, causal inferences cannot be made, and later-recognized symptoms were not captured. Despite these limitations, symptom-based screening, particularly incorporating symptoms such as muscle pain and taste loss, may help prioritize testing among HCWs. Implementing structured symptom checklists and routine monitoring could strengthen early detection and infection-control efforts in healthcare environments.

## Figures and Tables

**Table 1:** Association between selected variables and frequency of at least one positive test among 132 healthcare workers tested for SARS-CoV-2 in San Juan, Puerto Rico, from 20 July 2020 to 3 December 2020.

Characteristics	Workers	Positive Test		*p*	OR	95% CI
	N	N	%			
All	132	6	4.55	––	––	––
Sex						
Women	104	3	2.88	0.110	1.00	Reference
Men	28	3	10.71	––	3.98	0.50–31.58
Age						
21–30 years old	38	2	5.26	0.187	1.00	Reference
31–40 years old	31	3	9.68	––	1.91	0.20–24.34
41–50 years old	24	2	4.17	––	0.79	0.01–15.90
51–60 years old	30	0	0.00	––	0.50	0.00–6.74
61–70 years old	9	0	0.00	––	0.00	0.00–23.25
BMI						
Normal	38	1	2.63	0.462	1.00	Reference
Overweight	30	3	10.00	––	4.03	0.31–221.25
Obese	35	1	2.86	––	1.09	0.01–87.64
Not Available	26	1	3.85	––	1.47	0.02–119.15
Underweight	3	0	0.00	––	0.00	0.00–490.22
Occupation						
Nurse	50	1	2.00	1.824	1.00	Reference
Physicians	27	3	11.11	––	0.17	0.00–2.21
Maintenance crew	11	1	9.09	––	0.00	0.00–1.60
Technicians	3	0	0.00	––	0.00	0.00–643.48
Other ^1^	51	1	1.96	––	0.98	0.01–78.43
Any symptoms						
Yes	81	6	7.41	<0.001	1.00	Reference
No	51	0	0.00	––	Inf ^2^	0.76-Inf ^3^
No. symptoms						
1–2	39	0	0.00	<0.001	1.00	Reference
3–4	27	4	14.81	––	Inf ^2^	1.01-Inf ^3^
5–10	15	2	6.67	––	Inf ^2^	0.50-Inf ^3^
Specific symptoms ^4^						
Headache	50	5	10.00	0.200	3.56	0.67–26.38
Tiredness	49	4	8.16	0.190	3.60	0.68–26.70
Muscle pain	29	5	17.24	0.002	21.04	3.20–413.19
Throat pain	28	3	10.71	0.111	4.00	0.70–22.77
Cough	16	2	12.50	0.157	3.96	0.52–22.34
Diarrhea	15	1	6.67	0.522	1.60	0.08–10.91
Nasal congestion	9	1	11.11	0.400	2.95	0.15–21.49
Taste loss	8	3	37.50	0.002	24.20	3.72–165.47
Smell loss	6	2	33.33	0.024	15.25	1.76–108.84
Fever	5	2	40.00	0.016	20.50	2.25–165.21
Chills	7	1	14.29	0.287	3.93	0.19–30.24

Note: “Inf” (Infinity) indicates an odds ratio or confidence interval could not be estimated due to zero events in a group. These results come from univariable analysis. ^1^ Other includes administrative assistants, security officers, social workers, medical laboratory scientists, and image center personnel. ^2^ Inf is short for infinity, arises when the odds of an outcome never occur, i.e., no recurrences of positive SARS-CoV-2 virus. ^3^ Inf in the 95% CI (Confidence Interval) for an OR (odds ratio) means that it is infinitely large due to perfect prediction, which causes the confidence interval to be unbounded or not calculable. ^4^ For all symptoms, the reference category is “No” (symptom not reported).

**Table 2:** Adjusted and unadjusted Log-Odds for positive SARS-CoV-2 test by symptom count.

Number of Symptoms	Model 1			Model 2		
	Log-Odds (Unadj)	CI Lower (Unadj)	CI Upper (Unadj)	Log-Odds (Adj)	CI Lower (Adj)	CI Upper (Adj)
0	−4.34	−5.86	−2.83	−5.41	−7.69	−3.14
1	−3.91	−5.20	−2.63	−4.79	−6.71	−2.86
2	−3.49	−4.58	−2.39	−4.16	−5.77	−2.56
3	−3.06	−4.00	−2.11	−3.54	−4.87	−2.21
4	−2.63	−3.50	−1.75	−2.91	−4.05	−1.77
5	−2.20	−3.09	−1.30	−2.29	−3.36	−1.21
6	−1.77	−2.77	−0.77	−1.66	−2.82	−0.50
7	−1.34	−2.51	−0.17	−1.03	−2.40	0.33
8	−0.91	−2.30	0.47	−0.41	−2.05	1.24
9	−0.49	−2.10	1.13	0.22	−1.75	2.19
10	−0.06	−1.92	1.81	0.84	−1.48	3.17

## Data Availability

The data presented in this study is available as Table S1.

## Supplementary Materials

The following supporting information can be downloaded at: https://www.mdpi.com/article/10.3390/ijerph23010008/s1, Table S1. Metadata of 132 participants.
